# Physiotherapy Rehabilitation of a Conservatively Managed Patient With Pott’s Disease: A Case Report

**DOI:** 10.7759/cureus.33815

**Published:** 2023-01-16

**Authors:** Komal S Mandhane, Pratik Phansopkar, Neha V Chitale

**Affiliations:** 1 Physical Therapy, Ravi Nair Physiotherapy College, Datta Meghe Institute of Higher Education and Research, Wardha, IND; 2 Musculoskeletal Physiotherapy, Ravi Nair Physiotherapy College, Datta Meghe Institute of Higher Education and Research, Wardha, IND

**Keywords:** spinal tuberculosis, pott's disease-tuberculous spondylitis, sensory re-education, physiotherapy intervention, low-back pain (lbp), physical medicine and rehabilitation

## Abstract

The granulomatous disease tuberculosis (TB) is brought on by the bacteria *Mycobacterium tuberculosis*. Skeletal TB is involved in 10-35% of extra-pulmonary reported cases worldwide, with the most common kind being tuberculous spondylitis (Pott's disease). Depending upon the extent of spinal engagement, the clinical presentation may include back pain, discomfort, neurological abnormalities, as well as other clinical signs like temperature, malaise, and loss of weight. The multidisciplinary therapeutic care of Pott's illness is centered on a particular medicinal therapy, on-demand surgery, and a customized rehabilitation regimen. A 20-year-old female was diagnosed with Pott’s disease following investigations in Acharya Vinoba Bhave Rural Hospital (AVBRH), Sawangi (Meghe), Wardha, India. A tailored physiotherapy (PT) program was started and continued for six weeks, after which improvement in the mobility of the spine, pain relief, increased respiratory function, improved sensory function, and overall improvement in functional independence was markedly seen in the patient. The protocol included mobility, strengthening of lower limbs and core musculature, breathing, and postural correction exercises. Sensory re-education was done. Thus rehabilitation showed a great improvement in the patient and helped ease the patient's discomfort.

## Introduction

In impoverished or emerging nations, tuberculosis (TB) of the spine, also known as Pott's disease, affects 3 to 4% of tuberculous circumstances and 15% of extrapulmonary tuberculous conditions [1]. It is regarded as severe due to the great occurrence of neurological ailments and the impact of bone degradation, all the more so because the consequences are localized to the upper lumbar spine and/or lower thoracic spine in approximately 80% of patients [2]. It is commonly accepted that foci of bacilli trapped in the bone during the initial mycobacteremia of prime infection are the root of tuberculous osteomyelitis and arthritis. The principal focus (area) may be in the kidney or other viscera, or it may be active or dormant, visible or latent, in the lungs or in the mediastinum's lymph glands. Alternate routes for tuberculous bacilli to reach the spine include lymphatic outflow to the para-aortic lymph nodes or Batson's paravertebral venous plexus [3]. Early detection is crucial for preventing neurological sequelae and preventing the infection from spreading further. A particular medical intervention, on-demand operation, and a precise rehabilitative schedule form the basis of Pott's multidisciplinary therapeutic care.

The most prevalent and fatal type of TB infection is TB of the spine, often termed Pott's disease [4]. The spread of TB strains that have become resistant to particular treatments, hunger, drunkenness, insufficient TB control methods, filth, and increased travel appear to be factors in the increase in TB prevalence globally [3]. The gradual clinical picture that commonly precedes this pathology's appearance can lead to a hindered diagnosis. This infers the breakdown of the vertebrae, occasionally occurring kyphoses, as well as the spinal alignment issues it may cause [5]. The most frequent complications of TB of the spine include spinal deformity and paraplegia/quadriplegia. Kyphosis is often the result of TB of the dorsal spine, whereas lordosis reversal first occurs in the cervical and lumbar spines before kyphosis. Adults' kyphosis keeps getting worse when they receive conservative treatment and even after surgical treatment. The diaphragm is forced into the chest cavity and the rib borders move closer to the iliac crest, further worsening the breathing ability. Patients with severe thoracic kyphosis caused by TB may experience respiratory failure within a few years [6]. Spinal canal stenosis and deformity are caused by lacking diagnosis and treatment. Typically, thoracic TB of the spine involves the vertebral body and is localized there. The edges of the vertebrae, especially the anterior part of the vertebrae, are where tuberculous infection typically first manifests itself [7]. Over 70% of individuals having spinal TB develop a paraspinal abscess, which frequently occurs in concordance with epidural extension [8].

Limb weakness, gibbus, tenderness, and a palpable bump are the primary issues that occur. Patients who are in the active stage express fatigue, show weight loss, appetite loss, nocturnal sweats, and an increase in body temperature in the evening. The spine seems to have a regional kyphotic deformity which is sore to touch and rigid and uncomfortable to move. In order to treat patients with TB spine, goal-oriented medical as well as physiotherapy management is required. For Pott's disease, decompressive surgery followed by anti-TB chemotherapy is still the best treatment option. Medical management along with planned physiotherapy rehabilitation helps in easing the symptoms of the sick individual, timely recovery, and improved quality of life. The case of a 20-year-old woman who was recently diagnosed with Pott's disease is presented here. The patient experienced sensory loss in the lower extremity and low back pain. The patient was thoroughly assessed and a six-week physiotherapy program was formulated.

## Case presentation

A 20-year-old female from the Gondia district's Magardoh hamlet visited the Acharya Vinoba Bhave Rural Hospital (AVBRH), Sawangi (Meghe), Wardha, India with the chief complaints of diffused low back pain that had been plaguing her for the last two years and had crept up on her slowly at first. Additionally, the patient also reported a loss of sensation in the front and back of the distal lower limb. The patient gave a negative history of any other comorbidities. The patient had already been referred to a few private hospitals where she underwent a few investigations which revealed the presence of an abscess in the pre- and paravertebral space of the lower spine. After magnetic resonance imaging (MRI) and biopsy were carried out, the patient was diagnosed with TB of the spine L4-L5 and L5-S1. The patient was prescribed anti-TB medications and physiotherapy.

On observation

The patient was seen in a supine position with upper limbs by the side and lower limbs extended. An abscess was seen on the lower spine region.

On palpation

Grade 3 tenderness (patient winces and withdraws the affected part) was present on the lower lumbar region of the back. On examination range of motion (ROM) and manual muscle testing were taken (Tables 1 and 2).

**Table 1:** Pre-rehab Range of Motion Using Goniometer

Joint	Movement	Active (Right/Left)	Passive (Right/Left)
Hip	Flexion	0-55/0-60	0-60/0-65
	Extension	55-5/60-5	60-0/65-0
	Abduction	0-20/0-20	0-30/0-30
	Adduction	0-10/0-10	0-15/0-15
	External rotation	0-15/0-15	0-20/0-20
	Internal rotation	0-12/0-15	0-20/0-20
Knee	Flexion	0-110/0-110	0-120/0-120
	Extension	110-0/110-0	120-0/120-0
Ankle	Plantarflexion	0-15/0-15	0-15/0-20
	Dorsiflexion	0-10/0-10	0-15/0-15
Lumbar spine	Flexion	0-25	0-30
	Extension	0-10	0-20
	Lateral flexion	0-20/0-20	0-25/0-25
	Side rotation	0-20/0-20	0-25/0-25

**Table 2:** Pre-rehab Manual Muscle Testing by Kendall

Muscle Set	Right	Left
Hip flexors	2/5	2/5
Hip extensors	3/5	3/3
Knee flexors	3/5	3/5
Knee extensors	3/5	3/5
Ankle plantar flexors	4/5	4/5
Ankle dorsiflexors	4/5	4/5

Sensory examination

The assessment was done using American Spinal Injury Association (ASIA) impairment scale. All the motor functions were intact. There was a sensory deficit in the L5 and S1 segments with both pinprick and light touch impaired. Loss of sensation over the lateral and posterior calf and dorsum of the foot was noted.

Intervention

The main goal of physical therapy is to minimize the symptoms. The primary goal is to improve respiratory function, relieve back pain, increase muscle strength, increase ROM, improve sensory function, improve quality of life and prevent secondary complications.

Before initiating the treatment, it is essential to educate the patient about her condition, its complications, the prognosis, and the benefits physical therapy will have in easing her symptoms, relieving pain, and early recovery.

Strengthening exercises included isometrics initially, followed by resistive exercises first by manual resistance later using weights and mechanical resistance with 10-20 repetitions. Low back pain was managed with the application of transcutaneous electrical nerve stimulation (TENS) and moist heat. ROM exercises (both active and passive) up to full range were given to improve mobility. Mobility exercises for the trunk were given with proper assistance. Breathing exercises included pursed lip breathing and thoracic expansion exercises with five and ten repetitions respectively twice a day. Sensory re-education was done once a day. Postural correction techniques were taught. Aerobic exercise training was started in the fourth week using a static cycle and treadmill (Table 3).

**Table 3:** Physiotherapy Rehabilitation Protocol ROM: range of motion SLR: straight leg raise TENS: transcutaneous electrical nerve stimulation

Phase	Goal	Intervention
Day 1-week 1	I) To promote muscular endurance.	Isometric exercises of the upper and lower back, glutei muscles, hamstring, and quadriceps. Static abdominals with the back press were performed.
	II) To improve breathing.	Breathing exercises included pursed lip breathing and thoracic expansion exercises with an inspiratory hold of 5 seconds.
	III) To increase ROM.	Active ROM exercises to all joints of the upper and lower limbs. Heel slides, SLR, and dynamic quads were performed 10 times twice a day along with ankle-toe movements.
	IV) To relieve pain and spasm.	Application of heat pack before exercise therapy was done. Post-treatment 10 mins of cryotherapy using an ice pack. TENS was applied for lower limbs.
	V) Postural correction.	The patient was taught to sit upright. Shoulder shrugs, scapular retraction, and cat and camel exercises were given.
Week 2-4	I) To maintain muscle strength.	Isokinetic movements were given in the second week. It progressed to resistive exercises using manual resistance initially and later using weight cuffs of 0.5 kg for both upper and lower limbs. In the fourth week, the load was increased to 2kgs for both extremities.
	II) To improve breathing and increase functional capacity.	Pursed lip and thoracic expansion exercises were continued with increased respiratory hold.
	III) To increase and maintain ROM.	Active ROM exercises of all joints were continued. Exercises of week 1 with increased reps. Trunk bending exercises forward, backward, and sideways were started. Ambulation up to 100 meters was allowed.
	IV) To improve ambulation.	Ambulation was done under supervision. The patient was made to walk in the parallel bar to correct posture and gait pattern.
	V) To increase and maintain sensory function.	Sensory re-education was done. Different textured fabrics were used to stimulate sensory receptors.
	VI) To relieve low back pain.	Application of TENS to the lower back region along with moist heat to relieve pain and spasm.
Week 4-6	I) To increase and maintain muscle. strength.	Resistive exercises using weight cuffs of 2kg for upper and lower limbs. A head lift with the back press, abdominal curl-ups, diagonal curl-ups, and cat and camel position was performed.
	II) To improve breathing and increase aerobic capacity.	Pursed lip and thoracic expansion exercises were continued. Aerobic exercise training using a treadmill, stationary bicycle, stretching, running, and jogging was advised for at least 30-40 mins per day.
	III) To maintain ROM.	Active ROM exercises of all joints. Trunk bending exercises forward, backward, and sideways were continued. Front lunges, side lunges, and yoga pose for back stretching like cobra pose, extended child’s pose, pigeon’s pose, and reverse plank pose were performed. Ambulation up to 200 meters and more by six weeks was encouraged. Staircase climbing was performed under supervision.
	IV) To improve functional capacity and endurance.	Aerobic exercises using a treadmill, static cycle with continuous monitoring of vitals. Fast walking, jogging, and running to be incorporated in daily life.

## Discussion

One of the most debilitating effects of extra-pulmonary TB is Pott’s disease, often known as spinal TB [9]. Spondylitis brought on by TB is a rare condition that develops and progresses gradually [10]. Pott's disease is an ailment of the intervertebral discs between two consecutive vertebrae. Any system or tissue within the human body might get infected with TB when it starts in the lung. The spinal column is the extra pulmonary site that is most frequently affected [11].

The patient was sent to the physiotherapy department in the aforementioned case with the primary complaints of low back discomfort, sensory deficiency, and weakness in the lower limbs. On the complete assessment of the patient, a six-week rehabilitation protocol was made. The treatment included ROM exercises, pain reduction modalities, postural correction exercises, breathing exercises, and strengthening exercises along with sensory re-education. The effects of these exercises were remarkably seen post-treatment.

Rakesh Krishna Kovela and others conducted a study on the effectiveness of physiotherapy on the functions of lower limbs in a patient with Pott’s disease. The treatment plan included the application of TENS, breathing exercises, sensory re-education, and mobility exercises which by the end of the session showed marked improvement in the patient [10]. Another study by Zaoui and others on patients with Pott’s spine over a span of eight years also showed significant enhancement in the health of the patients. The rehabilitation protocol included initial immobilization of the affected spinal segment with the help of a corset, followed by mobility exercises, sensory integration, thoracic mobility exercises, ambulation, and ergonomic advice [2].

This case report shows the effects of vigorous physical therapy. The patient was able to function independently owing to an improvement in joint mobility and muscular strength. TENS stimulation and moist heat therapy were applied to alleviate low back pain and muscular spasms. Respiratory function was significantly improved post-exercise. The overall endurance and functional capacity of the patient showed notable improvement.

## Conclusions

A planned and goal-oriented physiotherapy regimen along with medications leads to early recovery and easing of symptoms of patients with Pott’s disease. Thus we conclude that the management specially designed for the patient was beneficial in increasing the mobility, stability, functional independence, and endurance of the patient thus improving the quality of life.

## References

[1] Waqas M, Qadeer M, Faiz F, Alvi MA, Bari ME (2015). Computed tomography-guided biopsy for Pott's disease: an institutional experience from an endemic developing country. Asian Spine J.

[2] Zaoui A, Kanoun S, Boughamoura H, Ben Maitigue M, Bouaziz MA, Khachnaoui F, Rejeb N (2012). Patients with complicated Pott's disease: management in a rehabilitation department and functional prognosis. Ann Phys Rehabil Med.

[3] Agrawal V, Patgaonkar PR, Nagariya SP (2010). Tuberculosis of spine. J Craniovertebr Junction Spine.

[4] Turgut M (2001). Spinal tuberculosis (Pott's disease): its clinical presentation, surgical management, and outcome. A survey study on 694 patients. Neurosurg Rev.

[5] Doe K, Lopes M, Taha S, Leriche B, Nogues L (2009). Neurosurgical management of paraplegia complicating Pott's disease: a six-case study [Article in French]. Neurochirurgie.

[6] Jain AK, Dhammi IK, Jain S, Mishra P (2010). Kyphosis in spinal tuberculosis - prevention and correction. Indian J Orthop.

[7] Griffith JF, Kumta SM, Leung PC, Cheng JCY, Chow LTC, Metreweli C (2002). Imaging of musculoskeletal tuberculosis: a new look at an old disease. Clin Orthop Relat Res.

[8] Rodriguez-Takeuchi SY, Renjifo ME, Medina FJ (2019). Extrapulmonary tuberculosis: pathophysiology and imaging findings. Radiographics.

[9] Khan D, Saddique MU, Paul T, Murshed K, Zahid M (2021). Metastatic adenocarcinoma of the lung mimicking miliary tuberculosis and Pott’s disease. Cureus.

[10] Chauhan I, Qureshi MI, Kovela RK (2022). Effectiveness of physiotherapy intervention on functional activities of lower limbs in a patient with Pott’s spine. J Med Pharm Allied Sci.

[11] Lakhwani M, Raipure A, Seth N, Phansopkar P (2022). Impact of multidisciplinary approach in managing pott spine - a case report. Med Sci.

